# The Institutional Worker

**Published:** 1918-08-17

**Authors:** 


THE
### INSTITUTIONAL WORKER
Being a Special Supplement to "The Hospital"

### OUR BUREAU OF INFORMATION.

### Rules for Correspondents.

1. Every letter must be accompanied by the coupon to be cut from the back cover (inside page) of The Hospital, current issue, and must contain the name and address of the correspondent with pseudonym for publication if desired. Replies by post cannot be given save under exceptional circumstances at the Editor's discretion.
2. Letters from Approved Homes in reply to special needs published in the Bureau should state terms and full particulars, and be sent prepaid under cover to the Editor of the Bureau with name written across coupon for identification.
3. Proprietors of Homes which have not yet been entered on the List of Approved Homes, but have spare accommodation likely to suit special needs, are invited to write for an application form for registration. The fee for registration, which includes two announcements of the Home in the Bureau and other privileges, is 10s.
4. All communications to be addressed to the Editor of The Hospital, 28 Southampton Street, Strand, London, W.C. 2, and marked "Bureau of Information."

### INSTITUTION FACTS AND FIGURES.

### Bedroom Suppers for Nurses Off Duty.

#### Answer to July Question.

The question was :

**In those hospitals having no kitchen in the Nurses' Home what arrangements can be made whereby the sisters and nurses who are off duty in the evening may have the comfort of supper in their bedrooms? What method may be adopted in order to avoid the dining-room maid being faced by a more or less depleted pantry when she wishes to lay breakfast? (The difficulty seems to be not so much as regards the food as the crockery.)**

#### "By Thorough."

The question of suppers in bedrooms was ever a vexed one and difficult to deal with. I gladly offer my experience in connection with the matter, and suggest two ways which I have found to work admirably.

Only sisters, staff nurses, and third-year nurses were allowed supper in their bedrooms, other grades of staff coming to the dining-room. The staff provide their own tray and crockery, which is kept in the ward-kitchen, pantry, or cupboard. The nurse requiring supper leaves out her tray, ready set, and the junior nurse on the ward takes it to the dining-room, the supper being served from the table by the person taking the meal, and the tray is then taken to the bedroom by the same nurse. In the morning the tray is carried back to the ward by the owner.

In this way no work or inconvenience is caused to any of the domestic staff, and the most junior nurse has the satisfaction of knowing that very soon her turn too will come to have supper in her bedroom.

The other alternative is as follows :—

The tray and crockery are supplied by the matron, a deposit of 5s. being required for the loan of same, the money being returned when the tray is returned intact to the matron. Missing or damaged articles are charged for according to their value. We had white crockery with distinctive coloured bands, using a different colour for different grades of staff. The care of the tray and arrangements for conveying suppers to rooms were the same as in the first suggestion. The same idea is admirable for breakfasts in bed on days off.

### The Staff of a Small Institution.

Some idea as to the staff allocated to a small institution is given in a report of the London County Council respecting a Home which was opened to provide accommodation for about eighty girls and women of the mentally deficient class.

The original staff consisted, in addition to the superintendent, of one needle-room matron (deputy superintendent), £30 a year, rising by £1 a year to £35, with board, lodging, washing, and uniform; one laundry matron, £30 a year, rising to £32; one kitchen matron, £30 a year, rising to £32; three housemaid-attendants, £20, rising to £25; one night nurse, £30; one manual training assistant, £100, rising to £150.

Experience soon showed, however, that the class of patient admitted required more than merely domestic care. Great difficulty was experienced in getting candidates for appointment as housemaid-attendant because of the unattractive title of the post, which tended to give a false impression of the duties attaching to it. The work which had to be done in the institution garden, moreover, proved to be so valuable for the patients that it was considered wise to develop it, and additional garden ground was secured. This involved the allocation of one member of the staff almost entirely to the supervision of outdoor work.

All these developments have necessitated a rearrangement of the staff, which it is proposed shall be as follows :—In addition to the night nurse, a charge nurse for day duty. It is not considered necessary that such nurse shall be certificated, but she should have had experience sufficient to enable her, under the direction of the superintendent, to undertake simple nursing duty as well as to look after the domestic work of the wards. She is to rank, for housework, with the three industrial matrons who have charge respectively of the kitchen, the laundry, and the needle-room.

The post of housemaid-attendant to be abolished, and instead, second-class nurses appointed, without previous experience, to whom definite training in the special care required by the mentally defective will be given in the institution.

One of the present housemaid-attendants who has shown special ability in that direction shall be allocated definitely to outdoor work.

In commenting upon these proposals the Asylums Committee remarks that for a total of eighty patients a total supervisory staff by day of eight does not seem to be excessive. At the time of the report the patients numbered sixty-three, and the salaries of the staff, including the superintendent, medical officer, dentist, chaplain, and clerical assistant, worked out at the rate of £524 a year, and the value of the emoluments at about £410. On a population of sixty-three patients, this means a cost of approximately £14 16s. 6d. a year per head, or 5s. 8½d. a week. This cost includes an allowance for the value of lodging, which does not represent actual expenditure in the same way as salary or cost of food.

Under the new scheme the salaries will be :—Charge nurse, £30 a year, rising to £32; second-class nurse, £20, rising to £24, and subsequently to £30; outdoor attendant, £30, rising to £32.

Having regard to the difficulty which is experienced in the Asylum Service in securing suitable candidates for appointment, the Asylums Committee feels it would be useless to offer less remuneration. The new scheme will involve a total salary list of £572, with £456 in respect of emoluments. On the basis of eighty patients this represents £13 a year, or 5s. per week per patient. At maximum salaries the total cost will reach £1,107 a year, or not quite 5s. 5d. a week for each patient.

### AUGUST QUESTIONS.

### 1. War Vacancies.

**Give model "Terms of Appointment" applicable for the substitute of a hospital's administrative official who has been called to the Colours. Provision to be made for the possible return to duty of the official previous to the termination of the war and the filling of the appointment in case of the non-return of the official concerned. Describe fully any experiences in this connection that may prove of value to hospital managers generally.**

### 2. Bedroom Suppers for Nurses Off Duty.

**In those hospitals having no kitchen in the Nurses' Home what arrangements can be made whereby the sisters and nurses who are off duty in the evening may have the comfort of supper in their bedrooms? What method may be adopted in order to avoid the dining-room maid being faced by a more or less depleted pantry when she wishes to lay breakfast? (The difficulty seems to be not so much as regards the food as the crockery.)**

#### RULES.

The following rules must be observed:—

1. Contributions must be written on one side of the paper. Brevity and terseness are desirable features. The MSS. must bear the name and address of the sender and be accompanied by coupon to be cut from the back cover (inside page) of the current issue of THE HOSPITAL. A pseudonym must be chosen if the name is not to be published.

2. Contributions must be addressed to the Editor of THE HOSPITAL, Institutional Worker Supplement, 28 & 29 Southampton Street, Strand, London, W.C. 2, should reach him before the end of the current month, and be marked in the left-hand corner "Facts and Figures."

**A minimum payment of Five Shillings will be made for each published answer.**

### QUESTIONS INVITED.

IN connection with our Question Box, we offer every month five shillings each for the two best questions which are sent in for consideration. The questions may be on any institutional subject and concern any department, from the secretary's to the porter's, or the matron's to the domestic staff; the one essential is that they must be practical and deal with points that have a definite relation to hospital or institutional administration, upkeep, finance, or management. Points relating to artificers' work, housekeeping, the laundry, out-patients, and other practical matters will be welcome. It is hoped that thus, when difficult or doubtful points arise in the course of their work, institutional workers will be encouraged to put them in the form of a question and send them to the Editor, THE HOSPITAL Bureau of Information, 28 & 29 Southampton Street, Strand, W.C. 2, marked "Question Box."

The best questions will be published in due course and our readers will be given the opportunity of answering them. Thus all institutional workers may be able to cooperate with the view of helping the work and smoothing the difficulties of each other. In short, we want the Question Box of the *Institutional Worker* to be freely used by every worker habitually.

### ENQUIRIES AND ANSWERS.

### *THE SICK AND IN NEED.*

#### Home for Child.

YOU might write to the Secretary of Dr. Barnardo's Homes, Stepney Causeway, E. 1, giving details of the child's birth, etc.; and to the Secretary of the Foundling Hospital, Guilford Street, Russell Square, W.C. 1, who may possibly help you in the matter.—W. A.

#### Home for Partially Blind Gentleman.

YOU might apply to the Indigent Blind Visiting Society, 8 Red Lion Square, W.C., for advice regarding admission to a home, or to the Incorporated Association for Promoting the General Welfare of the Blind, 258 Tottenham Court Road, W.—INTERESTED.

#### Home for Incurable Patient.

THE case you mention may possibly be admitted to either of the following homes: St. Joseph's Home for Incurables, Burlington Lane, Chiswick—a small weekly payment is required and admission is by selection; or St. Cyprian's Home for Incurables, 31 The Grove, Hammersmith, W. Application should be made to the Sister Superior in the first case, and to the Hon. Secretary of the second-named institution.—LONDON.

#### Pay Hospitals.

WE suggest that you make application to the Secretary, St. Andrew's Hospital, Dollis Hill, London, N.W. 2, which receives for treatment persons of limited means. You might also apply to the Hampstead General Hospital. The minimum fee in the pay wing at this institution is £2 2s. Medical attendance is not provided.—X. Y. Z.

### *MISCELLANEOUS.*

#### Help Offered.

YOU might offer your services to either of the following: The Metropolitan Asylums Board, Victoria Embankment, E.C. 4, who require, we believe, a number of women as nurses, wardmaids, kitchen- and scullery-maids; Lady Ampthill, of the Voluntary Aid Detachment, Devonshire House, W. 1, who may still have vacancies for women as cooks, motor-drivers, etc., not only for service in England, but also for France, Salonika, Malta, and Egypt.—IDA.

#### Splints.

NUMEROUS splints have been designed for the surgical treatment of gunshot injuries and fractures, and we have described several types in these columns. The essential principle involved is to secure the immobilisation of the fractured bones while leaving latitude for the proper dressing of wounds. An excellent type is a leg and thigh cradle splint designed by Major E. W. Hey Groves. This is a wire cradle splint and can be obtained from Messrs. Ferris and Co., Ltd., surgical-instrument makers, Bristol.—O. G. L.

### *WAR MATTERS.*

#### Bonus for Red Cross Nurse.

THE "war bonus" given to Red Cross Nurses by the Joint Committee is granted for one year's consecutive service from December 31, 1916, to January 1, 1918, with only the usual holiday during that period. We suggest that you write to the Matron-in-Chief, 83 Pall Mall, London, S.W. 1, and make inquiries.—RED CROSS.

#### Addresses Wanted.

THE addresses you ask for are: Scottish Women's Hospital, 2 St. Andrew's Square, Edinburgh; the Territorial Force Nursing Service, 80 Pall Mall, London, S.W. 1; Queen Alexandra's Imperial Military Nursing Service Reserve, War Office, Adastral House, Victoria Embankment, E.C. 4; The Wounded Allies' Relief Committee, 8 Grosvenor Gardens, S.W. 1.—BANFF.

#### Increased Allowances to Child of War Widow.

THE first child of men fallen in the war receives 6s. 8d. a week, the second 5s., and any others 4s. 2d. each. Relatives with the care of orphan children are entitled to 10s. a week for the first child and 9s. 2d. for all subsequent ones.—DISTRICT NURSE.

### *EMPLOYMENT AND TRAINING.*

#### Preliminary Training.

YOU are too young to enter a recognised training-school, and we suggest that you apply to any of the following institutions: The Eye Hospital, Bristol; Builth Cottage Hospital and Convalescent Home; Bristol Royal Hospital for Sick Children, St. Michael's Hill, Bristol; Llandrindod Wells Hospital. It is advisable to send in your application to the matron of the institution which you select quite six months before you wish to begin your training.—STROUD.

#### Training-Schools.

APPLY to the following institutions: Derbyshire Royal Infirmary, Derby; the North Staffordshire Infirmary, Stoke-on-Trent; Birmingham General Hospital; or the Wolverhampton and Staffordshire General Infirmary. Obtain "How to Become a Nurse," published by The Scientific Press, Ltd., 28 and 29 Southampton Street, Strand, W.C. 2, price 2s. 10d., post free.—MIDLANDS.

#### Training for Dispenser.

YOU might train in dispensing at any of the following institutions: Westminster College for Lady Dispensers, 112 St. George's Road, Southwark, S.E. 1; the Westminster College of Pharmacy, 100 Clapham Road, S.W. 9; the London School of Pharmacy, 7 Westbourne Park Road, W. 2; the School of Dispensing, 284 Streatham High Road, S.W. 16; or the South-Western Polytechnic Institute, Manresa Road, Chelsea, S.W. 11.—KATE.

